# Dentists and the Census Bill

**Published:** 1892-04

**Authors:** 


### Editorial, Etc.

DENTISTS AND THE CENSUS BILL.—A bill was introduced in Congress last week that, if passed, will have a direct bearing upon the completion of the bulletin of Baltimore manufacturing statistics.

As the Census law at present exists, there is a doubt as to whether the penalities for refusing to answer questions asked by the enumerators apply to all branches of statistics included within the scope of inquiry of the Census office, or solely to the population statistics. It is feared that prosecutions can be made only in those cases where citizens have refused to answer the questions put to them by the population enumerators, and that the agricultural, manufacturing and other divisions have no authority to enforce answers to such questions as may be asked by their enumerators. The bill referred to provides penalties for refusal to answer any questions included in the schedules of the Census Office, and will be used in the nature of thumbscrews and spiked boots to enforce replies.

The dentists of Baltimore, who, by their refusal to answer certain questions asked by the Manufacturing Division enumerators, have caused the delay in the work of preparing the bulletin of Baltimore's statistics of manufactures, will be the first victims of the inquisitorial machine, if the bill is passed. The Baltimore bulletin of manufactures cannot now be brought out before midsummer. Had these dentists answered the questions put them by the enumerators, the bulletin would have been issued last January.—*Baltimore American*, April, 1892.

This Journal published in the January number, 1881, the correspondence between Dr. T. B. Gunning and the Census Bureau. Dr. Gunning's clear and able letter convinced the Superintendent of the Census Bureau that the unwarranted demand for statistics on the ground that dental offices are "establishments of productive industry" was made in ignorance of what dentistry is. The information furnished by Dr. Gunning saved the profession generally from annoyance and trouble, as the Chief of the Census in 1880 (Gen. Francis A. Walker) determined to omit from the tabulations, "Dentistry in all its branches," when he learned of the protests of dental practitioners against making reports.

In August, 1890, agents of the Census Department distributed blanks to dentists in Baltimore and other cities to be filled with statistics of manufactures, which they claimed the law compelled the dentists to give. Dentists agreed not to answer the questions as they regarded dentistry a profession, and several dentists went from Baltimore to Washington to protest against their being classified as manufacturers and enumerated as such in the compilation of the statistics relating to Baltimore's manufactures. The chief of the manufacturer's division expressed himself to the effect that as some dentists "manufacture sets of teeth, plates and almost everything else but the raw material that they use in their business, these men are certainly manufacturers in the same sense that a blacksmith who makes a shoe from a bar of iron and fits it to the horse's hoof is a manufacturer."

As the "Philadelphia Press" very justly remarked at the time, although theology, law and medicine had by a prescriptive right monopolized the distinction 'learned professions,' "why the repair of teeth should come under mechanical employment and the repair of the body belong to a liberal profession is not easy to see. The subject has its difficulties and we cannot wonder that the attempt to classify has brought them to the surface."

Although as previously stated, Supt. Walker, in 1880, determined to omit "dentistry in *all* its branches from the tabulations for the 10th census, Supt. Porter, his successor in 1890, includes "plates, crowns, caps, &c., which are manufactured wholly or in part outside the mouth and afterwards inserted." "There can be no doubt," he claims, "that such features of the profession are clearly within the limits of the census inquiry relating to productive industries and as such are, under the law, to be included in the tables of manufactures."

The incorporated bodies of the city of Baltimore and of the State of Maryland in 1890 unanimously adopted resolutions declining to make returns to the Census Bureau: the Philadelphia County Dental Association, also in 1890, unanimously adopted resolutions instructing its "members to decline to attempt the impossibility of making any returns,"and in the New England States,it is reported that"fourteen (14) societies unanimously passed resolutions at a recent meeting in Boston that they would endorse the action of the Baltimore dentists."

